# Route patterns of the collateral venous pathway in patients with tumors invading the superior sagittal sinus: an angiographic study and clinical applications

**DOI:** 10.1007/s10143-024-02547-1

**Published:** 2024-08-09

**Authors:** Pawit Jirawisan, Sarun Nunta-aree, Bunpot Sitthinamsuwan, Ekawut Chankaew

**Affiliations:** 1 Division of Neurosurgery, Department of Surgery, Faculty of Medicine Siriraj Hospital, Mahidol University, 2 Wang Lang Road, Siriraj, Bangkok Noi, Bangkok, 10700 Thailand; 2 Department of Neurosurgery, Neurological Institute of Thailand, Bangkok, Thailand

**Keywords:** Angiography, Brain tumor, Collateral venous pathway, Diploic vein, Invasion, Occlusion, Parasagittal, Superior sagittal sinus

## Abstract

Chronic occlusion of the superior sagittal sinus (SSS) by tumors in the midsagittal region causes the collateral venous pathway (CVP). Understanding common patterns of CVP is helpful in reducing surgical complications. This study aimed to investigate the CVP found in patients with SSS-invading tumors, and to provide information on the prevention of operative venous complications. From January 2015 to December 2022, this retrospective study collected patients with tumors that invaded the SSS and underwent digital subtraction angiography of intracranial vessels. Data collected included sex, age, tumor pathology, tumor location along the SSS, tumor side, degree of obstruction of the SSS, types and route patterns of the CVP, and the distance between the tumor and the diploic vein (DV). Twenty patients (6 males, 14 females) were recruited. The prevalence of CVP types was 90% for DV, 35% for end-to-end anastomosis of superficial cortical vein, 15% for meningeal vein, and 20% for other types of CVP. The pteriofrontoparietal and occipitoparietal diploic routes were found on the cerebral hemisphere contralateral to the tumor significantly more than in the cerebral hemisphere ipsilateral to the tumor. Of all patients with presence of collateral DV, 61% had a very close (less than 1 cm) distance between the nearest DV and tumor attachment in the SSS. DV in the cerebral hemisphere contralateral to the tumor was the most common type of CVP found in patients with tumor-induced SSS obstruction. Most of the collateral DV was located very close to the SSS tumor attachment. Neurosurgeons should realize these findings when planning a craniotomy.

## Introduction

The superior sagittal sinus (SSS) is a major intracranial dural sinus that extends from the foramen cecum and drains into the torcular herophili [1, 2]. Slowly progressive obstruction of the SSS by the midsagittal tumor could lead to the development of the collateral venous pathway (CVP) that reroutes venous blood from the SSS to other dural venous sinuses [3, 4]. Parasagittal meningioma is a common brain tumor that causes this situation [5–7]. SSS occlusions have been found in approximately 50–57% of meningioma in the parasagittal area [8–10]. Other types of tumor associated with SSS occlusion have been reported in a limited number, for example, skull metastasis, malignant glioma, osteogenic sarcoma, and hemangiopericytoma [11–16]. The main treatment strategy for the aforementioned neoplasms with SSS invasion remains tumor resection with preservation of the main venous drainage.

Various types of CVP were reported in patients with SSS obstruction. Most reported types of CVP included end-to-end anastomosis of the superficial cortical vein (EEA of SCV), diploic vein (DV) and meningeal veins (MV) [17–19]. Other infrequent types of CVP were also reported, such as sinus pericranii and scalp veins [11, 17–21]. During surgery of tumors invading the SSS, care should be taken to avoid injury to CVP [22–25]. Inadvertent damage to this crucial venous structure can cause serious complications that may lead to operative morbidity and mortality, accounting for up to 10% and 3%, respectively [10, 23]. Venous complications related to surgery include brain edema, infarction, and intracerebral hemorrhage [26]. Therefore, neurosurgeons must know the functional anatomy and CVP of the intracranial venous structures [24, 27].

In the literature, there have been only a small number of studies on overall CVP in patients with tumor-induced SSS obstruction [17–19, 21]. Therefore, we conducted the present study to determine CVP in SSS-invading tumors to provide information to prevent venous complications and related operative adverse events.

## Materials and methods

### Patient population

The present study included consecutive patients who underwent surgery for the treatment of tumors that invaded the SSS at our institute from January 2015 to December 2022. All patients revealed midsagittal brain tumors attached to the SSS on preoperative cranial imaging and exhibited obstruction of the SSS to variable degrees on preoperative cerebral digital subtraction angiography (DSA). Individuals with previous cranial surgery or radiation therapy, vascular malformation, and unavailable DSA were excluded from the study.

### Ethical approval

The protocol for this study was approved by the Siriraj Institutional Review Board (SIRB), Faculty of Medicine Siriraj Hospital, Mahidol University, Bangkok, Thailand; Certificate of Approval (COA) number Si 373/2023. This study complied with the principles set forth in the 1964 Declaration of Helsinki and all of its later amendments or with comparable ethical standards.

### Consent to participate

Written informed consent to participate was not required for this retrospective study.

### Consent for publication

There was no personally identifiable data of research participants revealed in the present article. Written informed consent for publication was not required for this study.

### Data collection

Data from clinical history, imaging studies, and pathological reports were collected. The side and location of the tumor were determined by preoperative cranial magnetic resonance imaging (MRI). Tumor location was classified into 3 groups according to the area of attachment of the tumor to the SSS, including anterior 1/3 of the SSS (from the crista galli to the coronal suture), middle 1/3 (from the coronal suture to the lambdoid suture), and posterior 1/3 (from the lambdoid suture to the torcula herophili). The DSA was reviewed to define the degree of obstruction of the SSS and the CVP patterns. CVP patterns were evaluated by 2 neurosurgeons (P.J. and S.N.). In cases of angiographic disagreement, a consultation was conducted with an expert in neurointervention (E.C.) for a definitive conclusion. Furthermore, data of tumor size on contrast-enhanced cranial MRI were collected for investigation of association between CVP types, route patterns, and tumor size.

### Collateral venous pathway (CVP) of the superior sagittal sinus (SSS)

**Fig. 1:**
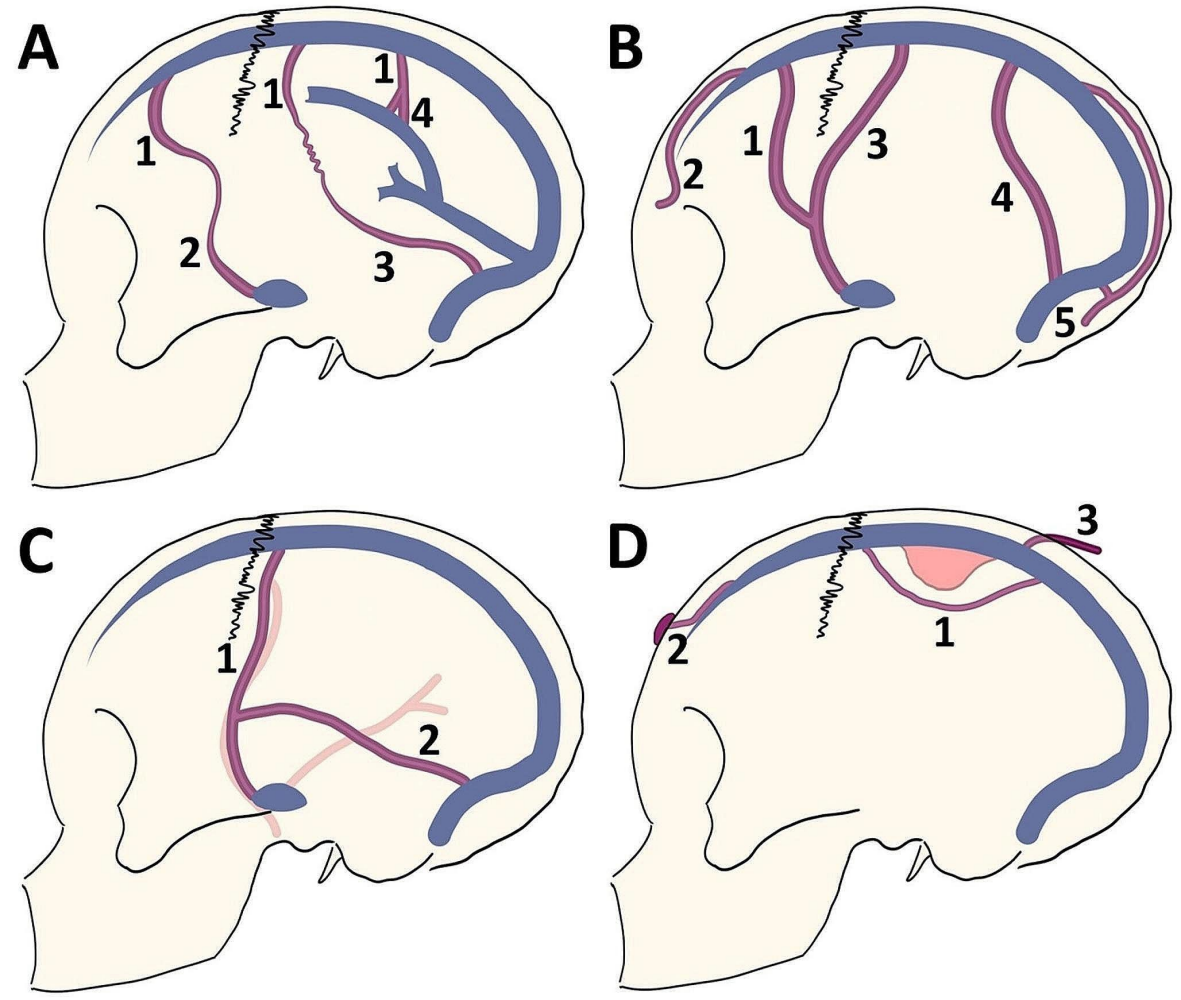
Schematic illustration of collateral venous pathway (CVP). (**A**) end-to-end anastomosis of superficial cortical vein (EEA of SCV), 1 = the superior sagittal group, 2 = the sphenoidal group, 3 = the tentorial group, and 4 = the falcine group; (**B**) diploic vein (DV), 1 = the pterional part of the fronto-orbital (PFO) route, 2 = the orbital part of fronto-orbital (OFO) route, 3 = the pteriofrontoparietal (PFP) route, 4 = the occipitoparietal (OP) route, and 5 = the occipitocervical (OC) route; (**C**) meningeal vein (MV), 1 = the superior sagittal sinus (SSS) draining into the sphenoparietal or cavernous sinus route, and 2 = the SSS draining into the transverse-sigmoid sinus route; and (**D**) other patterns of CVP, 1 = the SSS draining into the cortical vein making a detour around the area of SSS obstruction, 2 = the SSS draining into sinus pericranii, and 3 = the SSS draining into the scalp vein

The early to late venous phases of DSA were studied in an individual cerebral hemisphere to collect data on CVP. A CVP was defined as visible evidence of retrograde continuous flow of the venous channel extended from the SSS to other venous sinuses in both the anteroposterior and mediolateral dimensions in the DSA. CVP was categorized into 4 types, including (1) EEA of SCV, (2) DV, (3) MV, and (4) other CVP route patterns (Fig. 1). These types are described below.

**Fig. 2:**
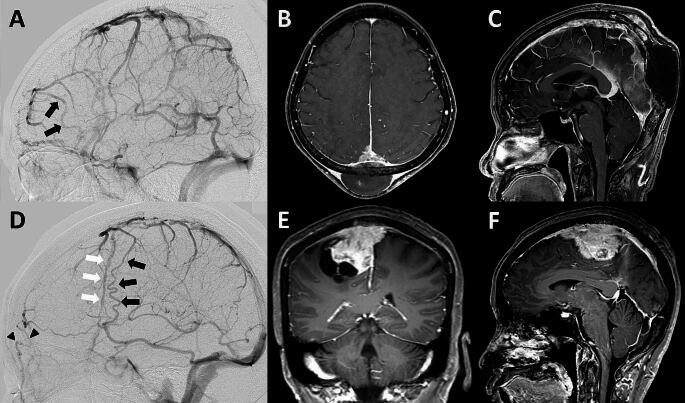
Route patterns of the EEA of SCV and DV. DSA and contrast-enhanced cranial MRI in case no.1 (**A-C**) and 2 (**D-F**). (**A**) EEA of SCV (black arrows) between the superior sagittal and sphenoidal groups on DSA; (**B, C**) contrast-enhanced cranial MRI in axial and sagittal views, pathological diagnosis was metastatic carcinosarcoma; (**D**) EEA of SCV (black arrows) between the superior sagittal and tentorial groups, OFO route of DV (black arrowheads), and PFP route of DV (white arrows) on DSA; and (**E, F**) contrast-enhanced cranial MRI in coronal and sagittal views, pathological diagnosis was meningioma. DSA, digital subtraction angiography; DV, diploic vein; EEA, end-to-end anastomosis; OFO, orbital part of fronto-orbital; PFP, pteriofrontoparietal; SCV, superficial cortical vein

**Fig. 3:**
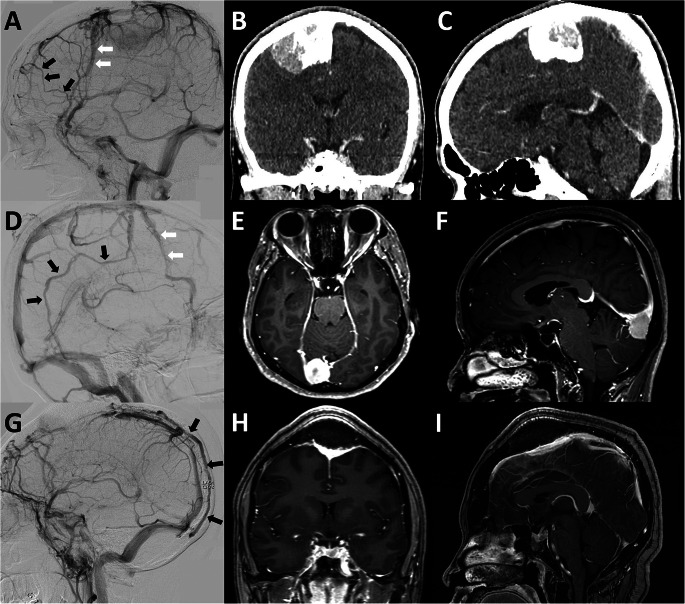
DV route patterns. DSA and brain imaging studies in case no.3 (**A-C**), 4 (**D-F**), and 5 (**G-I**). (**A**) PFO route of DV (black arrows), PFP route of DV (white arrows) on DSA; (**B, C**) contrast-enhanced cranial CT in coronal and sagittal views, pathological diagnosis was meningioma; (**D**) OP route of DV (black arrows), PFP route of DV (white arrows) on DSA; (**E, F**) contrast-enhanced cranial MRI in axial and sagittal views, pathological diagnosis was meningioma; (**G**) OC route of DV (black arrows) on DSA; and (**H, I**) contrast-enhanced cranial MRI in coronal and sagittal views, pathological diagnosis was meningioma. DSA, digital subtraction angiography; DV, diploic vein; OC, occipitocervical; OP, occipitoparietal; PFO, pterional part of fronto-orbital; PFP, pteriofrontoparietal

**Fig. 4:**
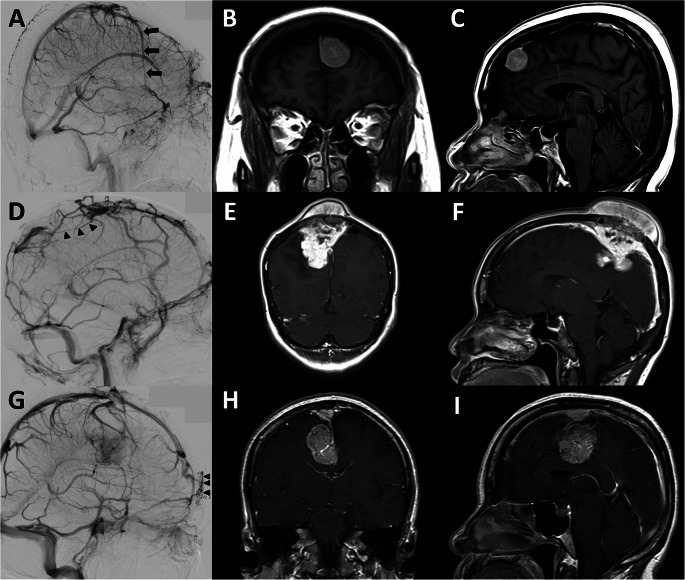
MV and other types of CVP. DSA and contrast-enhanced cranial MRI in case no.6 (**A-C**), 7 (**D-F**), and 8 (**G-I**). (**A**) MV (black arrows) accompanying the anterior branch of the MMA, running from the SSS to the sphenoparietal sinus on DSA; (**B, C**) contrast-enhanced cranial MRI in coronal and sagittal views, pathological diagnosis was solitary fibrous tumor; (**D**) cortical veins making a detour (black arrowheads) around the area of SSS obstruction on DSA; (**E, F**) contrast-enhanced cranial MRI in coronal and sagittal views, pathological diagnosis was metastatic adenocarcinoma; (**G**) sinus pericranii (black arrowheads) on DSA; and (**H, I**) contrast-enhanced cranial MRI in coronal and sagittal views, pathological diagnosis was meningioma. CVP, collateral venous pathway; DSA, digital subtraction angiography; MMA, middle meningeal artery; MV, meningeal vein; SSS, superior sagittal sinus

1. EEA of SCV (Fig. 2A and D). According to a study by Oka et al., the superficial cortical vein (SCV) can be divided into 4 route patterns based on its termination site. They included (1) the superior sagittal group that the SCV drains into the SSS, (2) the sphenoidal group that the SCV drains into the sphenoparietal or cavernous sinus, (3) the tentorial group that the SCV drains into the transverse or tentorial sinuses, and (4) the falcine group that the SCV drains into the inferior sagittal or straight sinus or its tributaries [17]. Therefore, in this article we analyze EEA of SCV based on its possible route patterns of venous drainage from the superior sagittal group, including the superior sagittal group draining to the sphenoidal group, the superior sagittal group draining to the tentorial group, and the superior sagittal group draining to the falcine group.
2. DV (Figs. 2D and 3A and D, and 3G). DV route patterns draining from the SSS were classified into 5 route patterns based on a study proposed by Tsutsumi et al. [28]. These subgroups included (1) the pterional part of the fronto-orbital (PFO), (2) the orbital part of the fronto-orbital (OFO), (3) the pteriofrontoparietal (PFP), (4) the occipitoparietal (OP), and (5) the occipitocervical (OC) routes. The PFO route is located in the anterolateral frontal area and drains into the middle meningeal vein (MMV) or sphenoparietal sinus. The OFO route is located in the supraorbital region and then drains into the subcutaneous veins adjacent to the supraorbital rim. The PFP route drains from the parietal segment of the SSS into the MMV or sphenoparietal sinus. The OP route drains from the SSS into the transverse-sigmoid junction. The OC route is an unpaired pathway that flows superinferiorly to the midline and eventually drains into the suboccipital venous plexus. In our individual patient, we also investigated the distance between the tumor and the closest DV origin in the SSS to gain insight into a safe craniotomy margin around the tumor.
3. MV (Fig. 4A). The MV runs along the meningeal artery and connects between the SSS and the dural sinus at the cranial base [29]. The two main route patterns were considered, including (1) the SSS draining to the sphenoparietal or cavernous sinus route and (2) the SSS draining to the transverse-sigmoid sinus route.
4. Other patterns of CVP (Fig. 4D and G). Other patterns of CVP had occasionally been reported in previous case studies [17, 20]. These CVP included the SSS draining into the cortical veins making a detour around the area of SSS obstruction, the SSS draining into sinus pericranii, and the SSS draining into the scalp vein.

### Evaluation of CVP in an individual cerebral hemisphere

CVP was evaluated with consideration of laterality, except for the OC diploic route pattern, which was an unpaired midline structure. On the basis of the side of the tumor, CVP on each cerebral hemisphere was designated as ‘the ipsilateral (same) side’ or ‘the contralateral (opposite) side’ of the tumor. In patients with tumors involving bilateral cerebral hemispheres, each hemisphere was defined as ipsilateral side.

### Statistical analysis

Demographic data was interpreted with descriptive statistics. Data were presented in numbers, percentages and mean ± standard deviation. The association between the presence of CVP and tumor-related factors was analyzed using Pearson’s chi-square test or Fisher’s exact test. A *p*-value of less than 0.05 was considered statistically significant. Data were analyzed with PASW Statistics version 27.0 (SPSS, Inc., Chicago, IL, USA).

## Results

Twenty patients were included in this study. Of them, 14 (70%) patients were female. The patient’s age ranged from 20 to 71 years. Parasagittal meningioma was the most common histopathological diagnosis. 90% of the patients had DV as CVP, while the prevalence of EEA of SCV, MV, and other CVP was 35%, 15%, and 20%, respectively. No statistically significant differences were defined regarding the proportion of patients with partial and complete SSS occlusion between the three tumor location groups (*p* = 0.571).

Of 20 patients, 40 cerebral hemispheres were obtained for angiographic study of the types and route patterns of CVP. Demographic characteristics are summarized in Table 1. A comparison between the ipsilateral (25 cerebral hemispheres) and contralateral (15 cerebral hemispheres) sides of the tumor did not show statistically significant differences in the presence of an individual CVP pattern. However, there was a trend towards a lower rate of the ipsilateral side to have collateral DV compared to the contralateral side of the tumor (64% vs. 93.3%, *p* = 0.060) (Table 2). The CVP route patterns were also compared according to the laterality between CVP and tumor (Table 3). The diploic routes of PFP and OP were significantly more likely to be found on the contralateral side than on the ipsilateral side of the tumor (86.7% vs. 52%, *p* = 0.040, and 40% vs. 8%, *p* = 0.036, respectively). Regarding other route patterns, there were no significant differences between both sides.

**Table 1:** Demographic characteristics (total *n* = 20)

Variable		Result
Sex, n (%)	Male	6 (30)
	Female	14 (70)
Age (years)	Mean ± SD	56.6 ± 14.1
Tumor pathology	Meningioma	15 (75)
	Metastasis	2 (10)
	Osteogenic sarcoma	2 (10)
	Solitary fibrous tumor	1 (5)
Tumor location, n (%)	Anterior 1/3 of SSS	2 (10)
	Middle 1/3 of SSS	13 (65)
	Posterior 1/3 of SSS	5 (25)
Side of tumor, n (%)	Right	13 (65)
	Left	2 (10)
	Bilateral	5 (25)
Degree of SSS obstruction	Partial occlusion	8 (40)
	Complete occlusion	12 (60)
Type of CVP, n (%)	EEA of SCV	7 (35)
	DV	18 (90)
	MV	3 (15)
	Other CVP	4 (20)
Route pattern of EEA of SCV, n (%)	Superior sagittal – sphenoidal	1 (5)
	Superior sagittal – tentorial	6 (30)
	Superior sagittal – falcine	0 (0)
Route pattern of DV, n (%)	PFO route	9 (45)
	OFO route	6 (30)
	PFP route	16 (80)
	OP route	7 (35)
	OC route	1 (5)
Route pattern of MV, n (%)	SSS – sphenoparietal/cavernous sinus	3 (15)
	SSS – transverse sinus	0 (0)
Other types of CVP, n (%)	Sinus pericranii	2 (10)
	Cortical venous detour	2 (10)

CVP, collateral venous pathways; EEA, end-to-end anastomoses; DV, diploic vein; MV, meningeal vein; n, number; OC, occipitocervical; OFO, orbital part of the fronto-orbital; OP, occipitoparietal; PFO, pterional part of the fronto-orbital; PFP, pteriofrontoparietal; SCV, superficial cortical vein; SSS, superior sagittal sinus

**Table 2:** Comparison of CVP types according to laterality between CVP and tumor

Type of CVPs	Laterality between CVP and tumor		*p*-value
	CVP located ipsilateral to the tumor (total cerebral hemispheres = 25)^a^	CVP located contralateral to the tumor (total cerebral hemispheres = 15)^b^	
EEA of SCV, n (%)	2 (8)	5 (33.3)	0.081
DV, n (%)	16 (64)	14 (93.3)	0.060
MV, n (%)	3 (12)	0 (0)	0.279

No variable with statistical significance (*p*-value < 0.05) in Table 2

^a^In some cerebral hemispheres, no CVP was seen

^b^In some cerebral hemispheres, several types of CVP could be seen

CVP, collateral venous pathway; DV, diploic vein; EEA, end-to-end anastomosis; MV, meningeal vein; n, number; SCV, superficial cortical vein

**Table 3:** Comparison of CVP route patterns based on laterality between CVP and tumor

Route pattern of CVP	Laterality between CVP and tumor		*p*-value
	CVP located ipsilateral to the tumor (total cerebral hemispheres = 25)^b^	CVP located contralateral to the tumor (total cerebral hemispheres = 15)^b^	
EEA of SCV, n (%)			
Superior sagittal – sphenoidal	0 (0)	1 (6.7)	0.375
Superior sagittal – tentorial	2 (8)	4 (26.7)	0.174
Superior sagittal – falcine	0 (0)	0 (0)	NA
DV, n (%)			
PFO route	7 (28)	5 (33.3)	0.722
OFO route	3 (12)	3 (20)	0.654
PFP route	13 (52)	13 (86.7)	0.040^a^
OP route	2 (8)	6 (40)	0.036^a^
MV, n (%)			
SSS – sphenoparietal/cavernous sinus	3 (12)	0 (0)	0.279
SSS– transverse sinus	0 (0)	0 (0)	NA

^a^*p*-value < 0.05 indicates statistical significance

^b^In some cerebral hemispheres, several types of CVP could be seen

CVP, collateral venous pathway; DV, diploic vein; EEA, end-to-end anastomosis; MV, meningeal vein; n, number; NA, not applicable; OFO, orbital part of fronto-orbital; OP, occipitoparietal; PFO, pterional part of the fronto-orbital; PFP, pteriofrontoparietal; SCV, superficial cortical vein; SSS, superior sagittal sinus

The prevalence of each type of CVP with respect to the location of the tumor attached to the SSS is shown in Table 4. The CVP types were not significantly different between the tumor location groups. A further comparison of route patterns based on tumor location was analyzed (Table 5). The result also did not show significant differences between the three tumor locations.

**Table 4:** Comparison of CVP types between tumor locations attached to the SSS

Type of CVP	Tumor location attached to the SSS			*p*-value
	Anterior 1/3 of SSS (total patients = 2)^a^	Middle 1/3 of SSS (total patients = 13)^a^	Posterior 1/3 of SSS (total patients = 5)^a^	
EEA of SCV, n (%)	1 (50)	4 (30.8)	2 (40)	0.837
DV, n (%)	1 (50)	12 (92.3)	5 (100)	0.276
MV, n (%)	1 (50)	2 (15.4)	0 (0)	0.502

No variable with statistical significance (*p*-value < 0.05) in Table 4

^a^In some patients, several types of CVP could be seen

CVP, collateral venous pathways; DV, diploic vein; EEA, end-to-end anastomosis; MV, meningeal vein; n, number; SCV, superficial cortical vein; SSS, superior sagittal sinus

**Table 5:** Comparison of CVP route patterns between tumor locations attached to the SSS

Type of CVP	Tumor location attached to the SSS			*p*-value
	Anterior 1/3 of SSS (total patients = 2)^a^	Middle 1/3 of SSS (total patients = 13)^a^	Posterior 1/3 of SSS (total patients = 5)^a^	
EEA of SCV, n (%)				
Superior sagittal – sphenoidal	0 (0)	0 (0)	1 (20)	NA
Superior sagittal – tentorial	1 (50)	4 (30.8)	1 (20)	0.732
Superior sagittal – falcine	0 (0)	0 (0)	0 (0)	NA
DV, n (%)				
PFO route	0 (0)	8 (61.5)	1 (20)	0.249
OFO route	1 (50)	4 (30.8)	1 (20)	0.732
PFP route	1 (50)	10 (76.9)	5 (100)	0.628
OP route	0 (0)	5 (38.5)	2 (40)	0.982
OC route	0 (0)	1 (7.7)	0 (0)	NA
MV, n (%)				
SSS – sphenoparietal/cavernous sinus	1 (50)	2 (15.4)	0 (0)	0.502
SSS – transverse sinus	0 (0)	0 (0)	0 (0)	NA

No variable with statistical significance (*p*-value < 0.05) in Table 5

^a^In some patients, several types of CVP could be seen

CVP, collateral venous pathway; DV, diploic vein; EEA, end-to-end anastomosis; MV, meningeal vein; n, number; NA, not applicable; OC, occipitocervical; OFO, orbital part of fronto-orbital; OP, occipitoparietal; PFO, pterional part of fronto-orbital; PFP, pteriofrontoparietal; SCV, superficial cortical vein; SSS, superior sagittal sinus

DV was the most common type of CVP in our study, and, naturally, it is vulnerable to being unintentionally injured during the craniotomy procedure for tumor resection. Therefore, in patients who demonstrated a collateral diploic venous pathway in the DSA, we measured the distance between the tumor and the closest DV origin in the SSS in lateral view of DSA. These distances were classified into three groups, including less than 1 cm, 1 to 2 cm and greater than 2 cm. The results are shown in Table 6. Additionally, the prevalence of individual type and route pattern of CVP with respect to the tumor size on contrast-enhanced cranial MRI is shown in Tables 7 and 8, respectively. There was no statistically significant association between CVP types or CVP route patterns and tumor size (5 cm of cut-off size).

**Table 6:** Distance between the nearest diploic vein and the tumor attachment on the SSS

Route pattern of the nearest DV (total *n* = 18)	Distance from tumor attachment on the SSS		
	< 1 cm	1–2 cm	> 2 cm
PFO route, n (%)	1 (5.6)	0 (0)	0 (0)
OFO route, n (%)	0 (0)	0 (0)	0 (0)
PFP route, n (%)	8 (44.4)	1 (5.6)	5 (27.8)
OP route, n (%)	2 (11.1)	0 (0)	1 (5.6)
OC route, n (%)	0 (0)	0 (0)	0 (0)
Total, n (%)	11 (61.1)	1 (5.6)	6 (33.3)

DV, diploic vein; n, number; OC, occipitocervical; OFO, orbital part of fronto-orbital; OP, occipitoparietal; PFO, pterional part of fronto-orbital; PFP, pteriofrontoparietal; SSS, superior sagittal sinus

**Table 7:** Comparison of CVP types according to tumor size

Type of CVPs	Tumor size		*p*-value
	≤ 5 cm (total patient = 10)^a^	> 5 cm (total patient = 10)^a^	
EEA of SCV, n (%)	2 (20)	5 (50)	0.350
DV, n (%)	10 (100)	8 (80)	0.474
MV, n (%)	1 (10)	2 (20)	1.000

No variable with statistical significance (*p*-value < 0.05) in Table 7

^a^In some patients, several types of CVP could be seen

CVP, collateral venous pathway; DV, diploic vein; EEA, end-to-end anastomosis; MV, meningeal vein; n, number; SCV, superficial cortical vein

**Table 8:** Comparison of CVP route patterns based on tumor size

Route pattern of CVP	Tumor size		*p*-value
	≤ 5 cm (total patient = 10)^a^	> 5 cm (total patient = 10)^a^	
EEA of SCV, n (%)			
Superior sagittal – sphenoidal	0 (0)	1 (10)	0.999
Superior sagittal – tentorial	2 (20)	4 (40)	0.628
Superior sagittal – falcine	0 (0)	0 (0)	NA
DV, n (%)			
PFO route	5 (50)	4 (40)	1.000
OFO route	1 (10)	5 (50)	0.141
PFP route	9 (90)	7 (70)	0.582
OP route	5 (50)	2 (20)	0.350
OC route	0 (0)	1 (10)	0.999
MV, n (%)			
SSS – sphenoparietal/cavernous sinus	1 (10)	2 (20)	0.531
SSS– transverse sinus	0 (0)	0 (0)	NA

No variable with statistical significance (*p*-value < 0.05) in Table 8

^a^In some patients, several types of CVP could be seen

CVP, collateral venous pathway; DV, diploic vein; EEA, end-to-end anastomosis; MV, meningeal vein; n, number; NA, not applicable; OC, occipitocervical; OFO, orbital part of fronto-orbital; OP, occipitoparietal; PFO, pterional part of the fronto-orbital; PFP, pteriofrontoparietal; SCV, superficial cortical vein; SSS, superior sagittal sinus

Regarding the procedure of tumor resection in this study, all of CVPs were completely preserved during the surgery. No venous structures functioned as CVP was intentionally or accidentally transected. There was no venous complication found.

## Discussion

Various types of CVP occur in patients with SSS obstruction caused by progressive gradual processes, such as tumor growth in the midsagittal region [17–21]. Knowledge of CVP is crucial for neurosurgeons in reducing the operative morbidity and mortality associated with the damage to these venous structures during tumor resection [10, 30–32]. However, the number of reports on CVP in patients with tumor-related SSS obstruction is scarce, and the modalities used to evaluate CVP, such as MRI [18, 21], magnetic resonance venography (MRV) [21], computed tomography venography (CTV) [18], virtual reality (VR) technology [33], DSA [17–19], 3-dimension venous model [21], venous ICG videoangiography [34], or any combination of these modalities, are varied. There are also discrepancies in the definition of CVP in imaging between studies. Furthermore, most previous studies focused only on a specific type of CVP [18, 19, 21]. Therefore, we conducted this study using the gold standard vascular imaging modality, DSA, to evaluate all possible CVP.

In our study, possible CVPs were divided into 4 types, including EEA of SCV, DV, MV, and other types of CVP. The majority (90%) of the patients had DV functioning as CVP. This result was comparable to a prevalence of 85.7% of DSA-confirmed DV reported in a previous study [18]. However, our results on prevalence of EEA of SCV (35%) and MV (15%) were much lower than previous reports (62.5–87.5%) [17, 19]. This could be because the distribution of the extent of involvement of SSS by the tumor and the dural sinuses of interest in these previous studies was different from ours.

The presence of each type of CVP was unaffected by the laterality between CVP and tumor and the location of the tumor along the SSS. However, there was a trend toward a higher rate of the presence of collateral DV in the cerebral hemisphere contralateral to the tumor than in the same side of the tumor (*p* = 0.060). This finding was somehow reflected in the significantly higher rate of the presence of diploic PFP and OP routes on the contralateral cerebral hemispheres compared to the ipsilateral cerebral hemispheres (*p* = 0.040 for the diploic PFP route pattern, and *p* = 0.036 for the diploic OP route pattern). Using contrast-enhanced MRI, a previous study did not find difference in the presence of DV between the side of the tumor and the contralateral side. Furthermore, the proportion of DV depicted by DSA was found to be increased when the SSS was occluded [18]. Even though DV exists in the presence or absence of the SSS-invading tumor, the proportion of blood flow-rich DV on DSA, increases with obstruction of the SSS. Regarding our study, DV, which functions as CVP, tends to develop on the contralateral side of the tumor. Our explanation was that the SSS-invading tumor may affect the development of collateral venous drainage in which the venous blood of the SSS was rerouted to the diploes in the contralateral cerebral hemisphere with respect to the tumor. However, it was difficult to evaluate whether the DV was not actually present on the tumor side because only DSA was used as the main modality to evaluate the presence of DV in this study.

### Clinical application

Surgical resection concurrently with preservation of venous flow or repair/reconstruction/bypass of the SSS is the mainstay treatment of tumors arising in the parasagittal region with SSS invasion [7, 8, 25, 31, 32, 35–39]. In some patients, combined microsurgery and radiosurgery are required to reduce resection-related morbidities and the risk of tumor recurrence [10, 40–44]. Occasionally, staged operations aiming to develop CVP in a period of time are a good alternative to prevent inadvertent venous complications [27].

**Fig. 5:**
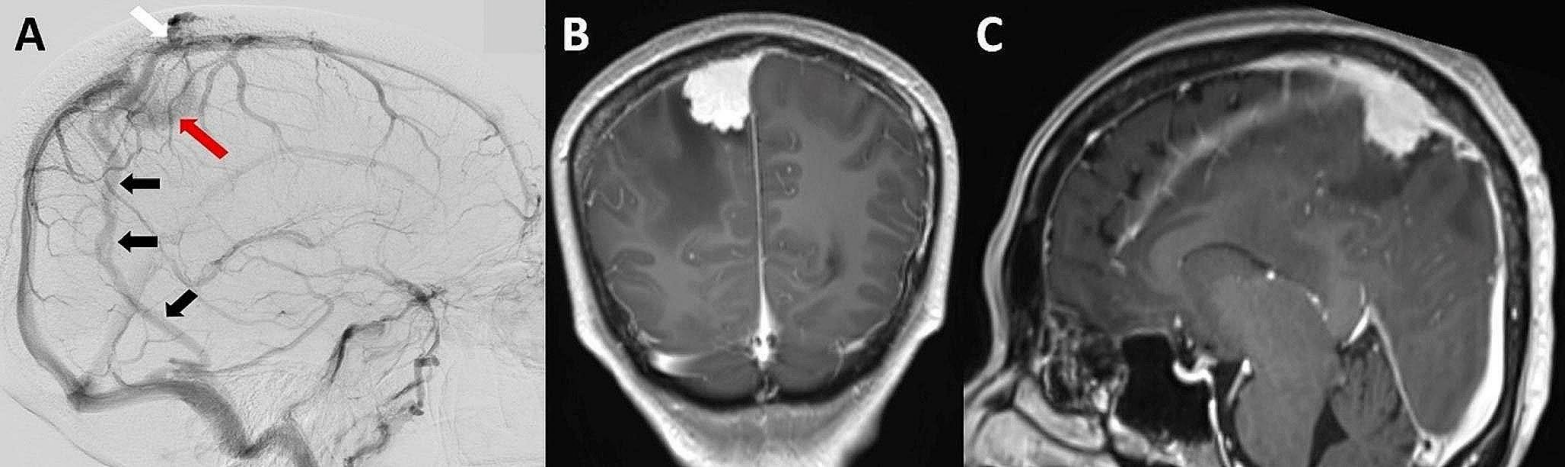
CVP with high risk of inadvertent injury during craniotomy. DSA and contrast-enhanced cranial MRI in case no.9 (**A-C**). (**A**) DSA showing an OP route of DV (black arrows) as the main CVP. The origin of the OP route of DV in the SSS is labeled as a white arrow, and the course of the OP route of DV is very close to the blushing of the tumor (red arrow); and (**B, C**) contrast-enhanced cranial MRI in coronal and sagittal views, pathological diagnosis was meningioma. CVP, collateral venous pathway; DSA, digital subtraction angiography; DV, diploic vein; OP, occipitoparietal route; SSS, superior sagittal sinus

To achieve adequate resection of parasagittal meningiomas, neurosurgeons tend to make the craniotomy size wider than the tumor margins. In studies by Sindou et al. [10] and Ricci et al. [9], the craniotomy to remove SSS-invading parasagittal meningioma was usually extended to approximately 2 to 3 cm beyond the margins of the occluded SSS and midline to reduce venous manipulation during tumor removal and provide adequate exposure for sinus reconstruction. However, a larger craniotomy could interrupt the collateral DV in the vicinity. If any of the functions of the DV are interrupted as the main CVP, this injury can lead to serious venous complications. Therefore, the distance of the diploic channels from the tumor attachment to the SSS is important for neurosurgeons in deciding the extent of craniotomy. In the present study, we explored the distance between tumor to the SSS and the collateral DV. The results were intriguing because more than 60% of patients with DV as CVP demonstrated very close proximity (less than 1 cm) to a DV from tumor attachment in the SSS. This finding should raise awareness of the careful planning of craniotomies in such cases. In surgery for tumors that cause SSS obstruction, we suggest performing preoperative neurovascular imaging in all cases for the detection of CVP that is vulnerable to injury during craniotomy, particularly DV in close proximity to the tumor (Fig. 5).

In our study, all of CVPs were completely preserved during tumor resection so that no venous complication was found. A previous study by Yamashiro et al. revealed a high transection rate of the DV in several locations of craniotomy approaches. Nevertheless, there was no venous complication reported in patients with transection of DV [45]. Regarding another study of Yamashiro et al., patients were divided into three groups based on venous sinus invasion by tumors. They included a group without venous sinus invasion, a group with SSS invasion, and a group with venous sinuses invasion other than the SSS. DVs were transected during craniotomy in 59 of 73 patients in the group without sinus invasion, and in 17 of 23 patients in the group with venous sinuses invasion other than the SSS. No new neurological deficits were reported in both groups. On the contrary, DVs were sacrificed in all of 14 patients in the group with SSS invasion. Two of five patients in this group who had transected early type of DV, developed new neurological deficits after the operations. Postoperative cranial MRI demonstrated focal brain edema caused by venous congestion [18].

Although adverse events after transection of DV are uncommon, it should be preserved as much as possible to avoid serious venous complications. Especially, transection of DV is contraindicated when it is only one major regional collateral venous drainage. We proposed surgical strategies for prevention of DV injury as the follows.

1. Limitation of extent of craniotomy.

- Utilization of surgical navigation system for identifying actual location of adjacent DV before craniotomy.
- Reduction in size of craniotomy; however, surgery may be more difficult and requires unusual trajectory or use of endoscopy for tumor resection.

2) In a situation when DV draining into scalp vein.

- Design of scalp incision appropriately and carefully.
- Meticulous dissection of the scalp during surgery.

3) In a situation when multiple DVs occupy on the ipsilateral side of tumor.

- Consideration of contralateral approaches, such as endoscopic contralateral interhemispheric transfalcine approach [46].

### Limitation

This study was a single institutional study with a small number of subjects included and may not be generalizable for clinical practice. Studies with larger sample sizes should be performed to provide a more accurate prevalence and nature of these CVPs. Furthermore, patterns of CVP may be changed according to patient position (supine or upright). Cerebral angiography was routinely performed in supine position, so that patterns of CVP in upright position could not be visualized. Regarding the last limitation, only DSA was used for identification of the venous structures and CVP in this study. Sometimes, it is difficult to specify accurate location of several types of veins, such as DV, MV, or scalp vein by using DSA alone. Contrast-enhanced MRI or computed tomography-digital subtraction venography (CT-DSV) would be useful to assess anatomical location of these venous structures.

## Conclusions

DV in the cerebral hemisphere contralateral to the tumor was the most common type of CVP found in patients with SSS obstruction due to meningiomas and other tumors originating in the parasagittal area. More than half of the collateral DV was located very close to the tumor. Neurosurgeons should be aware of this circumstance when planning a craniotomy.

## Data Availability

No datasets were generated or analysed during the current study.
